# Preterm functional outcomes are reflected in early postnatal proteome changes

**DOI:** 10.1038/s41390-025-04227-2

**Published:** 2025-07-17

**Authors:** Magdalena Zasada, Maciej Suski, Natalia Łapińska, Weronika Pogoda, Aleksandra Kowalik, Marta Olszewska, Przemko Kwinta

**Affiliations:** 1 Department of Pediatrics, Jagiellonian University Medical College, Krakow, Poland; 2 Department of Pharmacology, Jagiellonian University Medical College, Faculty of Medicine, Krakow, Poland; 3 Proteomics Laboratory, Centre for the Development of Therapies for Civilization and Age-Related Diseases CDT-CARD, Jagiellonian University Medical College, Krakow, Poland; 4 Department of Pharmaceutical Technology and Biopharmaceutics, Jagiellonian University Medical College, Faculty of Pharmacy, Krakow, Poland

## Abstract

**Background:**

Advances in omics technologies have enabled precise analysis of protein abundance. This study applies such methods to investigate urinary proteomic quantitative changes associated with prematurity.

**Methods:**

Urine samples were collected from very-low-gestational-age (VLGA) infants (*n* = 29) without premature brain damage, as assessed using the Kidokoro scale and magnetic resonance imaging at term-equivalent age, and from full-term infants (*n* = 19) on the 1^st^, 2^nd^, 3^rd^, 4^th^, 6^th^ and 8^th^ days of life. SWATH-MS analysis of the urine proteome, combined with bioinformatics, was utilized for the identification of regulated urinary proteins and altered functional pathways.

**Results:**

We identified 61 proteins that were significantly differentially abundant in urine throughout the study. The regulated urinary proteins were enriched in functional domains related to the immune system, hemostasis, and complement and coagulation cascades, indicating underdevelopment in VLGA infants. Conversely, the augmented pathways included extracellular matrix organization, cholesterol metabolism and PPAR signaling.

**Conclusions:**

The urinary proteome of VLGA infants differed significantly from that of term neonates, revealing protein profiles linked to immune system immaturity and hemostasis, altered metabolism and perturbed extracellular matrix metabolism. This study underscores how prematurity affects the urinary proteome, offering insights into the molecular pathways influenced by premature birth.

**Impact:**

- The urinary proteome of premature newborns differs from the urinary proteome of full-term newborns; analysis of urine proteins indicates the functional consequences of prematurity.
- In our study, we tested urine on the 1^st^, 2^nd^, 3^rd^, 4^th^, 6^th^ and 8^th^ days of life, which increased the reliability of the data.
- Examination of the urinary proteome at the first week of life allows us to demonstrate the functional consequences of prematurity.

**Category of study**: basic science

## Introduction

Almost eleven percent of infants are born prematurely^1^, and the level of immaturity plays a crucial role in determining the infant’s health^2^. In particular, infants born at <32 weeks of gestational age (very low gestational age, VLGA) have higher rates of morbidity and mortality than do full-term infants^3^. The first week of life is crucial not only for the adaptation of immature organs and systems to the extrauterine environment, but also for the occurrence of several complications of prematurity.

The urinary proteome is the full set of proteins present in urine. Urinary proteins are composed of kidney-derived (70%) and plasma-derived (30%) proteins^4^. Therefore, analysis of the urinary proteome can provide information about kidney-related disorders as well as about the conditions affecting distant organs^5^. The collection of a urine sample is simple, noninvasive, and free from clinical risk.

SWATH-MS is an emerging powerful acquisition methodology that enables the confident identification and reproducible quantification of several hundred proteins in every urine sample^5^.

Studies of the urinary proteome in infants born preterm are limited, and little is known about the influence of gestational and postnatal age on the expression of urinary proteins and their potential clinical relevance in this group of patients. Using a targeted approach, Charlton et al. identified several markers of renal development^6^. Starodubtseva et al. investigated the urine proteome of preterm infants with respiratory pathologies^7^. In a group of premature infants with the subsequent development of bronchopulmonary dysplasia (BPD), urinary proteome changes reflect alterations in protein abundances in the blood or respiratory tract^8^. Sylvester et al. identified seven urine proteins capable of providing highly accurate diagnostic and prognostic information for infants with suspected necrotizing enterocolitis^9^. Urinary proteomics has also been applied for the prediction of cardiovascular sequelae in preterm infants^10^. However, most of these studies were based on the analysis of a urine sample collected at a single time point. To our knowledge, SWATH-MS-based proteome exploration of VLGA infant urine collected frequently during the first week of life in the context of prematurity has not previously been performed.

The objective of this study was to perform comprehensive analyses of the urinary proteome in VLGA infants without major cerebral complications of prematurity with term-born controls to broaden our understanding of the pathophysiologic consequences of preterm birth manifesting within the first week of life.

## Methods

### Study groups

This single-center, prospective study was conducted in the Neonatal Intensive Care Unit (NICU) at the Institute of Pediatrics of Jagiellonian University Medical College in Cracow, Poland. Between April 2021 and July 2023, two cohorts of infants were consecutively and simultaneously enrolled:

1) VLGA newborns with a GA of 22 0/7–31 6/7 weeks admitted to the NICU within 0–24 h of life

2) Newborns with a GA ≥ 37 0/7 weeks with mild adaptation to extrauterine life problems (for example, transient tachypnea of the newborn) admitted to the NICU within 0–24 h of life.

The exclusion criteria for both cohorts were as follows: (1) major congenital anomalies of the heart/kidney or any structural abnormalities on cranial ultrasound (cUS) upon admission, (2) multiple pregnancies, and (3) clinical suspicion of metabolic/genetic disorders.

All VLGA infants underwent brain MRI at term-equivalent age (TEA), as previously described^11^. Brain MR images were categorized by two independent investigators according to the Kidokoro scale^12^. VLGA infants with a global brain abnormality score (GBAS) indicating normality composed the study group (*n* = 29). The control group consisted of all recruited full-term newborns (*n* = 19).

### Urine sampling and preparation

Urine samples for proteome analysis were collected at six time points, on days of life (DOLs) 1, 2, 3, 4, 6, and 8. Urine was collected noninvasively using sterile cotton balls (Paul Hartmann, Pabianice, Poland) placed in a disposable diaper, from which the urine was aspirated with a sterile syringe (B. Braun Medical AG, Sempach, Switzerland) and transferred to a sterile polystyrene test tube (FL Medical, Torreglia, Italy). In the case of urine collection for medical reasons via a sterilized urine bag (Zarys, Zabrze, Poland), some of the urine obtained was aspirated from the bag into a sterile vial using a sterile syringe. Any urine sample contaminated with stool was discarded, and a new sample was collected. Upon collection, the urine was immediately transferred to the Department of Clinical Biochemistry, Institute of Pediatrics, JUMC. The urine was concentrated by centrifugation for 10 min at 2600 × *g* at 4 °C in a Vivaspin Turbo 4 Centrifugal Concentrator filter (MWCO 3 kDa; Sartorius, Göttingen, Germany) and stored at −80 °C until use.

### Sample preparation for LC-MS/MS analysis

Prior to proteomic analysis, the collected urine samples were purified by acetone precipitation. Six volumes of ice-cold acetone were mixed with the urine sample, vortexed, and centrifuged at 14,000 × *g* for 10 min at 4 °C after overnight incubation at −20 °C. The protein pellets were then dissolved in buffer containing 2% SDS and 50 mM DTT in 0.1 M Tris-HCl (pH 7.6), vortexed, incubated at 95 °C for 5 min and clarified by centrifugation at 14,000 × *g* for 10 min. Prior to protein digestion, the total protein concentration was determined by the WF assay^13^. Next, 70 µg of total protein was transferred to Microcon-30 kDa centrifugal filter units (Merck, Darmstadt, Germany), denatured with 8 M urea in 0.1 M Tris-HCl (pH 8.5) and digested to peptides using the filter-aided sample preparation (FASP) protocol^14^. Briefly, proteins were alkylated with iodoacetamide and cleaved with LysC-trypsin mix (Promega, Madison, WI) at an enzyme‒protein ratio of 1:35. The digestions were carried out overnight in 50 mM Tris-HCl (pH 8.5) at 37 °C. After digestion, the peptide yields were determined by the WF assay, and aliquots containing equal amounts of total peptides were desalted on 96-well Oasis HLB 96-well µElution plates (Waters, Milford, MA). The samples were then concentrated to a volume of approximately 5 µL. For project-specific spectral library preparation, equal amounts of peptides from all of the samples included in the analysis were combined and subjected to a fractionation protocol. HpH fractionation on C_18_ Micro Spin Columns (Harvard Apparatus, Holliston, MA) was performed in 50 mM ammonium formate buffer (pH 10) with 12 consecutive injections of eluent buffer, comprising 5, 10, 12.5, 15, 17.5, 20, 22.5, 25, 27.5, 30, 35 and 50% acetonitrile in 50 mM ammonium formate buffer (pH 10), collected by centrifugation (300 × *g*, 2 min) and dried in a vacuum concentrator (Eppendorf, Hamburg, Germany). In this way, the peptides were distributed across 12 HpH fractions and analyzed by LC-MS/MS in DDA acquisition mode for library generation. Before the analysis, all samples and library peptide fractions were solubilized in 0.1% formic acid at a concentration of 0.5 µg/µl and spiked with the iRT peptide mix (Biognosys, Schlieren, Switzerland) for normalization of the retention time.

### Liquid chromatography-tandem mass spectrometry

For spectral library preparation, the peptide fractions (1 µg) were injected into a nanoEase M/Z Peptide BEH C18 75 µm i.d. × 25 cm column (Waters, Milford, MA) via a trap column nanoEase M/Z Symmetry C18 180 µm i.d. × 2 cm column (Waters). For library generation, each peptide fraction was separated using a linear gradient of phase B from 1% to 50% for 98 min (phase A - 2% ACN and 0.1% FA; B phase - 80% ACN and 0.1% FA) operating at a flow rate of 300 nL/min on an UltiMate 3000 HPLC system (Thermo Scientific, Waltham, MA) and applied to a TripleTOF 6600+ mass spectrometer (Sciex, Framingham, MA) operating in DDA acquisition mode. The main working parameters of the nanoelectrospray ion source (Optiflow, Sciex, Framingham, MA) were as follows: ion spray voltage, 3 kV; interface heater temperature (IHT), 200 °C; ion source gas 1 (GS1) 10; and curtain gas (CUR) 25. For DDA acquisition, spectra were collected in full scan mode (350–1400 Da), followed by one hundred CID MS/MS scans of one hundred most intense precursor ions from the preceding survey full scan exceeding 100 cps intensity under dynamic exclusion criteria. Individual urine samples (5 µg) were analyzed in SWATH acquisition mode in the microflow chromatography regime and separated using a nonlinear gradient (A phase - 2% ACN and 0.1% FA; B phase - 80% ACN and 0.1% FA): 0–3 min, 1% B; 3–7 min, 1–8% B; 7–27 min, 8–25% B; 27–36 min, 25–40% B; 36–43 min, 40–60% B; 43–44 min, 60–99% B; 44–51 min, 99% B; 51–52 min, 99–1% B; and 52–60 min, 1% B, with a flow rate of 5 μL/min. The main working electrospray ion source (Optiflow, Sciex, Framingham, MA) parameters were as follows: ion spray voltage, 4.5 kV; ion source gas 1 (GS1), 15; and curtain gas (CUR), 20. For SWATH acquisition, the spectra were collected in full scan mode (400–1250 Da), followed by one hundred SWATH MS/MS scans using a variable precursor isolation window approach, with m/z windows ranging from 6 to 90 Da.

### Mass spectrometric raw data analysis, spectral library generation and SWATH quantitation

The DDA data were searched against the human UniProt database (release 2022_1, 20,375 entries) supplemented with MaxQuant contaminants (245 entries) using the Pulsar search engine implemented in Spectronaut software (Biognosys, Schlieren, Switzerland)^15^ with default parameters ( ± 40 ppm mass tolerance at the MS1 and MS2 levels, mutated decoy generation method, and trypsinP enzyme specificity). Deep learning-assisted iRT regression was used as the iRT reference strategy for RT-to-iRT calibration, with the minimum R^2^ set to 0.8. The peptide, protein and PSM FDRs were set at 1%. The library was generated using 3–6 fragment ions per precursor.

The project-specific library was then used to analyze the SWATH data in Spectronaut (Biognosys, Schlieren, Switzerland). The data were filtered with a 1% FDR at the peptide and protein levels, while quantitation and interference correction were performed at the MS2 level. Protein grouping was performed via the ID picker algorithm^16^. The label-free quantitation (LFQ) was done by means of the Quant 2.0 algorithm, the Spectronaut standard for DIA data based on Top3 proteome quantitation method. For each identified protein group top three stripped peptide sequences were selected and averaged. For every precursor quantity estimation, the top three fragment ions were selected and averaged. The data were normalized by a global regression strategy, where a single reference analysis was constructed by taking the median peak intensity for 10,000 precursors profiles selected by Spectronaut, ranked by level of completeness. Linear regression normalization was then performed on this constructed reference by applying least squares regression. Based on the linear regression equation new values were predicted for each analysis, taking both intercept and slope of the regression line into account. Statistical testing for differential protein abundance was performed using t tests with multiple testing correction after Storey^17^.

Functional grouping and pathway annotations were performed using ClueGO^18^ in the Cytoscape 3.7.2 software environment^19^ with the use of PINE software^20^. We restricted the analysis to include only those regulated proteins, which were altered in at least 50% of the analyzed timepoints (minimum 3 out of 6). Human disease (release 17.02.2020), CORUM-3.0 (release 03.09.2018), KEGG (release 17.02.2020), REACTOME (release 17.02.2020) and WikiPathways (release 17.02.2020) ontologies/pathways were used in the analysis. The enrichment results were validated by enrichment/depletion two-sided geometric statistical tests with the Bonferroni step-down *p* value correction method. The minimum and maximum GO levels were set at 1 and 4, respectively, with the cluster criterion of a minimum of 5 genes constituting a minimum of 2% of the GO term. The kappa score threshold was set as 0.4.

### Statistical analysis of the clinical data

Qualitative values were compared using Fisher’s exact test. The Wilcoxon test was used to compare continuous variables. Differences were found to be statistically significant if the probability of the type I error alpha value was lower than 0.05. JMP® version 17.1.0 (JMP Statistical Discovery) was used for all of the statistical analyses.

## Results

The demographic and hospitalization data of the patients are presented in Table 1.

**Table 1:** Cohort demographics, course of hospitalization and diagnoses of all patients in urine proteomics study.

	Term-born neonates (*n* = 19)	Preterm neonates (*n* = 29)	*p*-value and test used
Characteristics of the current pregnancy			
Antenatal corticosteroid prophylaxis, *n* (%)	0 (0)	19 (65.5)	**<0.0001 **^**F**^
Maternal diabetes (any), *n* (%)	2 (10.5)	2 (6.9)	0.9999 ^F^
Maternal hypertension (any), *n* (%)	0 (0)	5 (17.2)	0.1418 ^F^
Maternal hypothyroidism, *n* (%)	3 (15.8)	1 (3.5)	0.2864 ^F^
Neurological disease, *n* (%)	1 (5.3)	0 (0)	0.3958 ^F^
Urinary tract infection, *n* (%)	6 (31.6)	0 (0)	**0.0022 **^**F**^
Infection (excluding urinary tract infection), *n* (%)	2 (10.5)	6 (20.7)	0.4511 ^F^
Smoking, *n* (%)	1 (5.3)	2 (6.9)	0.9999 ^F^
Alcohol use, *n* (%)	0 (0)	0 (0)	**-**
Infant’s medical history			
Gestational age (weeks; median [Q1;Q3])	39 [37;40]	30 [27.5;30]	**<0.0001**^**W**^
Birth weight (g; median [Q1;Q3])	3300 [2 895; 3 820]	1300 [995; 1530]	**<0.0001**^**W**^
Sex (female, *n* (%))	7 (36.84)	15 (51.72)	0.3820 ^F^
Pregnancy; median [Q1; Q3]	2 [1;4]	2 [1;3]	0.7336^W^
Delivery; median [Q1; Q3]	2 [1;3]	2 [1;3]	0.7483^W^
Mode of delivery: cesarean section/vaginal delivery, *n* (%)	11 (57.89) /8 (42.11)	24 (82.76) /5 (17.24)	0.0959 ^F^
Apgar score (5^th^ min; median [Q1;Q3])	10 [8;10]	7 [6;7.75]	**<0.0001**^**W**^
Nasal cannula use (days; median [Q1;Q3])	0 [0;1]	6 [0;22.5]	**0.0271**^**W**^
CPAP use (days; median [Q1;Q3])	0 [0;0]	0 [0;3]	**0.0426**^**W**^
Mechanical ventilation (days; median [Q1;Q3])	0 [0;0]	0 [0;5]	**0.0088**^**W**^
Duration of parenteral nutrition (days; median [Q1;Q3])	2.5 [1;3.25]	10 [6.5;13.5]	**<0.0001**^**W**^
Early-onset sepsis, *n* (%)	1 (5.26)	0 (0.0)	0.3958 ^F^
Late-onset sepsis, *n* (%)	0 (0.0)	2 (6.9)	0.5115 ^F^
Persistent ductus arteriosus requiring surgical treatment, *n* (%)	0 (0.0)	0 (0.0)	-
Intraventricular hemorrhage grade 3 or 4, *n* (%)	0 (0.0)	0 (0.0)	-
Periventricular leukomalacia, *n* (%)	0 (0.0)	1 (3.5)	0.9999 ^F^
Bronchopulmonary dysplasia moderate or severe, *n* (%)	0 (0.0)	2 (6.9)	0.5115 ^F^
Retinopathy of prematurity requiring treatment, *n* (%)	0 (0.0)	6 (20.69)	0.0684 ^F^
Necrotizing enterocolitis, *n* (%)	0 (0.0)	2 (6.9)	0.5115 ^F^
Acute kidney injury, *n* (%)	0 (0.0)	0 (0.0)	-

^W^– Wilcoxon test, ^F^- Fisher’s exact test.

The statistical tests used are indicated by F for Fisher’s Exact Test, W for the Wilcoxon rank-sum test, and *p*-values for each comparison are provided accordingly.

The bold values indicate statistically significant differences.

**Fig. 1:**
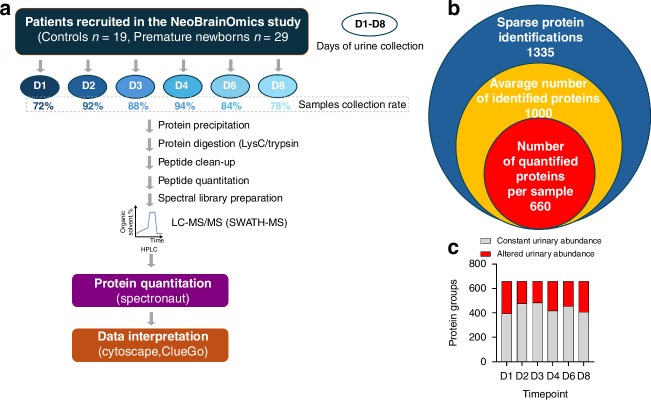
Overview of proteomic measurements in neonatal urine. Schematic description of the study design and the label-free proteomic workflow that included the SWATH acquisition methodology (**a**), which allowed for the reproducible quantification of several hundred proteins in every urine sample (**b**). Notably, the percentage of significantly differentially abundant proteins fluctuated during newborn observation but remained similar at all timepoints of urine collection (**c**).

The study design scheme and the proteomic analysis workflow are shown in Fig. 1a.

**Fig. 2:**
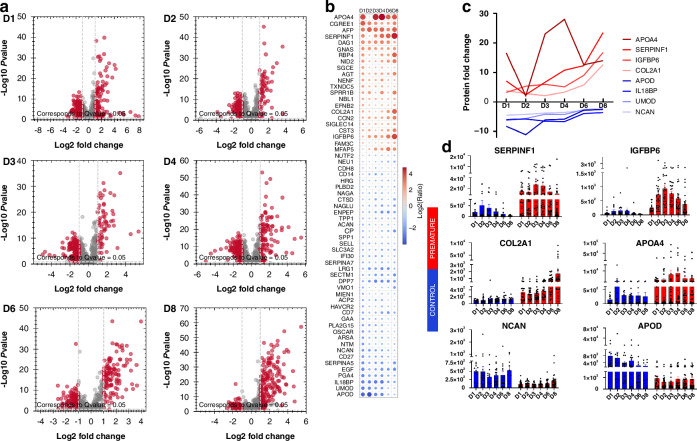
Regulated urinary proteins and their quantitative time-dependent changes. The evaluation of the composition of urinary proteins by SWATH proteomics revealed substantial differences between premature and term-delivered newborns at all timepoints (**a**). Sixty-one proteins were significantly regulated throughout the course of the study, showing a continuing trend of downregulation or upregulation during the first eight days of life (**b**). However, when analyzed in more detail, several of these proteins exhibited time-dependent quantitative trajectories of progressive normalization or deviation from the control group (**c**). Protein quantity analysis. The data (normalized MS intensity) are presented as bars (means ± SEMss) with individual values; *n* = 10–18 in the control group (blue) and *n* = 24–29 in the premature group (red). All presented data are statistically significant (*q* < 0.05) (**d**).

DDA mass spectrometry measurements resulted in the identification of 7400 proteotypic peptides, which allowed for the preparation of a spectral library comprising 1608 protein groups. The library was then used to analyze SWATH data with Spectronaut, resulting in the identification of 1335 protein groups at least once throughout the sample cohort (sparse profiles). On average, 1000 proteins were identified in each urine sample. Next, to create a robust and reliable quantitative dataset, we selected only those proteins that were identified in at least 80% of the samples by at least two unique peptides and performed a missing value imputation using a global imputation strategy where the missing values were imputed on the basis of random sampling from a distribution of low-abundance signals taken throughout the entire experiment. This resulted in the generation of a dataset of 660 proteins quantified in each sample (Fig. 1b). The estimated significant absolute fold change cutoff was set at 2.0 to ensure the power of the statistical analysis (statistical power ranging from 0.853 to –0.944, depending on the comparison). On average, 220 protein groups (ranging from 176 to 265) differed in urinary abundance on each collection day (Fig. 2a) and were similar across the study timepoints (Fig. 1c, Supplementary Tables S1–S7).

The majority of quantitatively significantly different urinary proteins exhibited a persistent difference between preterm infants and their corresponding full-term controls throughout the study period. (Fig. 2b). However, for several of them, time-dependent quantitative trajectories can be distinguished (Fig. 2b, c). For example, pigment epithelium-derived factor (SERPINF1), collagen alpha-1(II) chain (COL2A1) and insulin-like growth factor-binding protein 6 (IGFBP6) tended to increase in relative urinary abundance over time compared with those in the control group (Fig. 2b, c). During the study period, the concentrations of SERPINF1 and IGFBP6 exhibited a decreasing trend in the preterm group, as indicated by protein quantity changes (Fig. 2d). Consequently, the observed relative increase in their abundance is attributed to a concurrent reduction in urinary levels of these proteins in term controls at corresponding time points. In contrast, COL2A1 represents a distinct quantitative trait, wherein the relative difference between the experimental groups is primarily driven by changes in the preterm group (Fig. 2d). Conversely, neurocan core protein (NCAN) and apolipoprotein D (APOD) demonstrate a trend toward normalization of their relative urinary abundance over time (Fig. 2b). However, these trends result from distinct patterns of quantitative their changes during the study period. Specifically, NCAN exhibits a gradual increase in the preterm group towards the values observed in the term controls. In contrast, the normalization of APOD is driven by a time-dependent decline in urinary abundance among control newborns rather than by alterations in the preterm group (Fig. 2d).

**Fig. 3:**
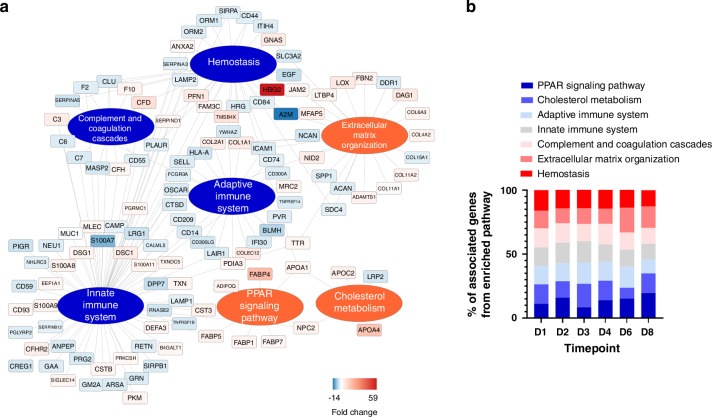
Functional pathway enrichment of regulated urinary proteins. Induced (red) and repressed (blue) functional pathways inferred from quantitative changes in the urinary proteome (**a**). The indicated protein quantitative fold change values were calculated as the means for all timepoints. The percentage of associated proteins from the enriched pathways remained constant at all timepoints assayed, indicating a similar functional landscape of differences between term and premature newborns during the first eight days of life (**b**).

Finally, we performed pathway analysis to elucidate the potential functional consequences of prematurity, which can be inferred from changes in the urinary proteome. As a result, we obtained a complex network of mutually interconnected processes (Fig. 3a) that were similarly altered throughout the experimental time course, as evidenced by the constant percentage of associated proteins identified in these pathways at all included timepoints (Fig. 3b).

The key functional domains enriched by regulated urinary proteins were those related to the maturation of the innate and adaptive immune system, hemostasis, and complement and coagulation cascades, all of which can be inferred to be underdeveloped in premature newborns (Fig. 3a).

In contrast, the upregulated pathways elicited from the urinary proteome included extracellular matrix organization, cholesterol metabolism and peroxisome proliferator-activated receptor (PPAR) signaling (Fig. 3a).

## Discussion

In this study, we explored the urinary proteome of VLGA infants to enhance our understanding of the functional consequences of prematurity present in their early postnatal period, a time when prematurity-related complications may begin or emerge. Our study holds significance, as frequent sampling in a consistent manner during the first week of life enhances the reliability and robustness of the results obtained. Through daily sampling during the first four days of life, supplemented by additional collections on the 6th and 8th days, we were able to clearly delineate physiological processes that are altered in preterm infants.

### Regulated urinary proteins and their quantitative time-dependent changes

We identified 61 proteins that exhibited significant changes in urinary abundance throughout the entire course of the study, showing either a stable difference or a continuing trend of down- or upregulation during the first eight days of life. Interestingly, the identified urine proteins exhibited varying patterns of concentration changes during the first week of life. Our study demonstrated that for certain proteins, the dynamics of urinary concentration changes over time in VLGA patients differ from those observed in full-term newborns.

One of the most upregulated proteins in the urinary VLGA proteome was APOA4. APOA4 is a multifunctional protein involved in lipid transport with antioxidant and anti-inflammatory properties. Our observation is similar to the results of Cabral et al., who also revealed urinary upregulation of proteins related to lipid metabolism in preterm infants in comparison with their term-born peers^10^. Interestingly, a comparison of apolipoprotein profiles between preschool children born at term or preterm revealed that the apolipoprotein A-I, A-IV, C-II, and C-III levels are significantly higher in the latter group^21^. We presume that higher APOA4 levels and the lack of a clear decrease in APOA4 concentration in the first week of life in premature infants compared with full-term neonates may facilitate digestion and lipid transport in immature neonates and may help regulate lipid balance and inflammatory responses in their intestinal mucosa.

PEDF/SERPINF1 is a multifunctional protein that has anti-angiogenic, anti-inflammatory, antioxidant, and neurotrophic effects. It also plays a significant role in maintaining and regulating microvascular homeostasis^22^. Moreover, PEDF has a high affinity for the collagen of the extracellular matrix^23^. PEDF plays a role in bone homeostasis by inhibiting bone resorption and increasing matrix mineralization^24^.

Insulin-like growth factor-binding protein 6 (IGFBP6) is involved in IGF signaling, and IGF is a major regulator of fetal growth and development in most organs^25^. After very preterm birth, the serum concentration of IGF-1 decreases^26^. We presume that the slower decrease in the rate of IGFBP6 in VLGA infants may be associated with their immaturity and the stronger influence of IGF on their growth. Similarly, Charlton et al. reported significantly higher urinary levels of IGFBP6 in preterm infants (median gestational age 34 weeks) at birth than in full-term infants. However, by 6 months of age, the urinary level of IGFBP6 was indistinguishable between the term and preterm groups^6^.

Neurocan (NCAN) is a component of the extracellular matrix in the brain and has a vital role in neuronal migration and plasticity, especially in the development of the cortex^27^. In a study by Leifsdottir et al., a lower level of NCAN was found in the CSF of preterm infants with adverse neurological outcomes^28^.

Apolipoprotein D (APOD) is implicated in modulation of the oxidative stress, inflammation, and the transport of small hydrophobic molecules. Interestingly, Starodubtseva et al. observed a lower level of APOD in the urine of neonates with infectious respiratory pathologies than in healthy neonates or neonates with respiratory difficulties of noninfectious origin^7^.

### Functional pathway enrichment of regulated urinary proteins

To obtain a broader context, we explored the functional pathway enrichment associated with only the regulated proteins that were changed on at least half of the sampling days. According to our study, the complement and coagulation cascades are repressed in VLGA infants. We identified the serine protease inhibitors alpha-1-antichymotrypsin (SERPINA3) and protein C inhibitor (SERPINA5), as well as prothrombin (F2) and mannose-binding lectin serine protease 2 (MASP2) as having lower urine concentrations than term-delivered newborns do. Interestingly, MASP2 is a member of the collectin protein family that can cleave prothrombin to form thrombin, exhibits thrombin-like activity and participates in complement cascade regulation^29,30^. These changes are accompanied by a reduction in the urinary CD55 level, which binds to complement proteins and accelerates their decay, disrupting the cascade^31^. We also observed the repression of the pathways enriched with proteins involved in the innate and adaptive immune systems. Psoriasin (S100A7), one of the most downregulated proteins in the urine of VLGA infants, is an antimicrobial peptide that exhibits potent activity against *E. coli*^32^. Due to its role in transporting immunoglobulins to mucosal surfaces, the polymeric immunoglobulin receptor (PIGR) is fundamental for effective mucosal immunity^33^. Osteoclast-associated immunoglobulin-like receptor (OSCAR) is involved not only in bone metabolism but also in the interaction between osteoclasts and other components of the immune system^34^. The increased abundance of cystatin C (CST3) in the urine of VLGA infants may reflect poor reabsorption by tubular cells due to immature proximal tubules or a smaller number of renal proximal tubules. Our results are in accordance with those of Kamianowska et al. who also reported higher cystatin C urinary excretion in stable, premature neonates than in their term-born peers^35^.

Moreover, VLGA infants exhibited repression of the hemostasis pathway. For example, we observed downregulation of alpha-2-macroglobulin (A2M), a multifunctional protein that plays a regulatory role in hemostasis by inhibiting proteases involved in coagulation and fibrinolysis^36^.

Collectively, the observed quantitative protein changes in the urine of prematurely born newborns indicate a decline in the functionality and efficiency of the immune, hemostatic, and coagulation systems.

Among the augmented pathways, the peroxisome proliferator-activated receptor (PPAR) signaling pathway is of particular interest as PPAR induction can ameliorate neuroinflammatory networks in the central nervous system^37^. Adiponectin (ADIPOQ) was upregulated in preterm urine. ADIPOQ is one of the most important hormones in insulin sensitivity and homeostasis. Interestingly, according to Smit et al., its levels are lower in the cord blood of SGA infants than in that of AGA infants^38^.

Fatty acid-binding protein 4 (FABP4) participates in regulating the neonatal glucose balance. Circulating FABP4 promotes hyperglycemia and stimulates hepatic glucose production by regulating the expression of key gluconeogenic enzymes^39^. Notably, the highest levels of FABP4 in blood were observed in neonates experiencing hypoglycemia^40^, and prematurity is a risk factor for hypoglycemia^41^.

Interestingly, fatty acid binding proteins (FABPs) in urine have been proposed as early predictive biomarkers of kidney injury^42^. Furthermore, Yamamoto et al. reported that the induction of FABP in proximal tubule cells reduces oxidative stress during hypoxia^43^.

Taken together, the upregulated proteins in the urine of the VLGA are enriched in processes that can represent regulatory signaling initiated to counteract functional deficiencies associated with preterm birth.

According to our results, during the first week of life, VLGA infants also present a urinary overrepresentation of proteins that are enriched in the extracellular matrix organization pathway. We believe that the increased expression of proteins from this pathway may indicate immature regulation of extracellular matrix proteins as they are more abundant in the urine of premature infants. Alternatively, it may indicate impaired maturation of tissue structures. The increased abundance of various collagen subunits may reflect the status of the increased turnover of bone, cartilage, and the extracellular matrix. However, perturbed extracellular matrix metabolism has been linked with the development of bronchopulmonary dysplasia. Interestingly, the level of protein-lysine 6-oxidase (LOX), which plays a key role in regulating the stability of the extracellular matrix, was elevated in the oxygen-injured lungs of newborn mice and infants with BPD or who are at risk for BPD^44^. The LOX level was more abundant in the urine of VGLA infants than in that of term-born infants in our study.

### Limitations

To minimize differences between the study groups other than the maturity of the newborn, we compared premature babies with the best brain MRI results (according to the Kidokoro scale) with the healthiest full-term newborns who were admitted to our NICU. However, although the patients in our preterm group were “typical” premature infants, the full-term neonates were not completely healthy, which may have influenced the observed results. Also, we did not adjust the obtained results for specific clinical conditions or interventions that may differentiate our groups and could potentially influence protein expression in the urine. Furthermore, as we did not collect additional tissues or body fluids from term and preterm neonates, we were unable to perform systematic protein identification analyses to comprehensively characterize the background proteome specific to our study conducted in newborns. Consequently, pathway enrichment analysis based on the whole human proteome may have been less directly associated with the processes related to preterm birth. Moreover, as this was an exploratory study, the research hypothesis was not defined.

## Conclusions

This study offers new evidence of time-dependent changes in the urinary proteome of VLGA infants during the early postnatal period, a critical phase in which the physiological sequelae of prematurity begin to emerge. Intensive urine sampling across the first eight days of life revealed 61 proteins with significant alterations in comparison to the term-born control, involved in key pathways such as immunity, coagulation, lipid metabolism, growth regulation, and extracellular matrix organization. Our findings highlight a repression of immune and hemostatic pathways in preterm neonates, suggesting functional immaturity. Conversely, proteins linked to lipid and glucose metabolism, as well as extracellular matrix turnover were upregulated, likely reflecting adaptive responses to prematurity. The identification of specific proteins (e.g., APOA4, PEDF, IGFBP6, FABP4) underscores the potential of urinary proteomics as a non-invasive tool for assessing organ development and identifying early markers of prematurity-related complications. Further research in larger cohorts is needed to confirm these findings and evaluate their clinical relevance.

## Supplementary information

Supplementary Tables S1–S6

Supplementary Table S7

## Data Availability

All the data analyzed during this study are included in this article. Further inquiries can be directed to the corresponding author.
